# Safety, efficacy, and selection strategy of laparoscopic local gastrectomy for gastrointestinal stromal tumors in the esophagogastric junction

**DOI:** 10.3389/fsurg.2022.1015126

**Published:** 2022-09-27

**Authors:** Haiqiao Zhang, Xiaoye Liu, Zhi Zheng, Jie Yin, Jun Zhang

**Affiliations:** Department of General Surgery, Beijing Friendship Hospital, Capital Medical University, Beijing, China

**Keywords:** esophagogastric junction, gastrointestinal stromal tumors, surgical method, local gastrectomy, gastroesophageal reflux

## Abstract

**Objective:**

To investigate the safety, efficacy, and selection strategy of laparoscopic local gastrectomy for stromal tumors in the esophagogastric junction.

**Methods:**

Thirty-eight patients with mesenchymal tumors in the esophagogastric junction were retrospectively enrolled from April 2018 to July 2021 in which the upper edge of the tumor is less than 2 cm from the Z-line or has invaded the Z-line <1/2 circumference. Surgical outcomes, complications, recover, and postoperative gastroesophageal reflux of both groups were compared.

**Results:**

27 patients underwent wedge resection, and 11 underwent resection by opening all of the layers of the stomach wall. Operative time (90.0 vs. 181.8 min, respectively, *P* = 0.001) was shorter for the WR group vs. RASW. Blood loss (20 vs. 50 ml, respectively, *P* = 0.012) was less for the WR group vs. RASW. Recovery of the RASW group was slower in terms of time to pass gas (2 vs. 3 days, *P* = 0.034), time to oral intake (2 vs. 4 days, *P* = 0.007), time to semi-liquid food intake (4 vs. 8 days, *P* = 0.003), and postoperative hospitalization (5 vs. 8 days, *P* = 0.001) vs. WR. In terms of short-term complications (≤30 days), no significant between-group differences were observed. Cardia stenosis did not occur in either group. In the WR group, one patient experienced mild reflux at 6 months and recovered 1 year after surgery. In the RASW group, one patient experienced severe gastroesophageal reflux at 6 months and 1 year after surgery, which was not entirely relieved by taking antacids. No other patients have gastroesophageal reflux.

**Conclusion:**

Laparoscopic local gastrectomy is safe and feasible for mesenchymal tumors in the esophagogastric junction in which the upper edge of the tumor is less than 2 cm from the Z-line or has invaded the Z-line <1/2 circumference, and has achieved an excellent short-term effect. The choice of surgery is based on the relationship between the tumor and the position of the cardia.

## Introduction

Gastrointestinal stromal tumors (GISTs) are the most common mesenchymal tissue-derived malignancies in the digestive tract, accounting for 1%–3% of all gastrointestinal malignancies, and are more likely to occur in the stomach (50%–70%) (1, 2). For GISTs with tumor diameter less than 2 cm, endoscopic treatment is preferred, while for tumor diameter larger than 2 cm, surgery is the main method of treatment (3). The goals of surgical therapy mainly include ensuring the integrity of the tumor capsule and achieving negative tumor margins. GISTs are primarily implanted in the abdominal cavity and metastasize *via* the blood, and lymph node metastasis is rare. Therefore, there is no need for lymph node dissection during the operation (4). With the widespread adoption of laparoscopic technology in the surgical field, laparoscopic local gastrectomy (LLG) has been safely and effectively applied for the surgical treatment of GISTs (5). Lukaszcryk first reported laparoscopic gastrectomy for GISTs in 1992 (6). With the increasing maturity of minimally invasive techniques, studies have found that laparoscopic technology is safe and feasible for GISTs (7, 8) and has now become the preferred method. It can significantly reduce tissue bleeding and damage, fully expose the surgical field of vision, improve the safety of the operation, shorten the hospital stay, and reduce postoperative discomfort. Currently, the most commonly used laparoscopic surgery methods for the treatment of GISTs include four types: wedge resection (WR), resection by opening all of the layers of the stomach wall (RASW), mucosa-preserving resection, and proximal gastrectomy. The main factors that affect the choice of gastric stromal tumor surgery are the size and location of the tumor (9), which have a significant impact on the perioperative outcome. However, such studies have primarily focused on the body of the stomach and the antrum, and there are few studies on the esophagogastric junction (EGJ) (10).

The incidence of GISTs in the EGJ is low, accounting for 5.8%–13.5% (11–13), so there are few reports on this topic. The anatomy of GISTs in the EGJ is complex, and the operation and functional retention are difficult. Therefore, proximal gastrectomy was often used for GISTs in EGJ in the past. However, the high incidence of postoperative gastroesophageal reflux seriously affects the quality of life of patients (14). Complications such as cardiac stenosis may occur after local gastrectomy. Therefore, there is currently no way to reconstruct the digestive tract that has been widely recognized. Xiong et al. (15) found that GISTs in the EGJ in which the upper edge of the tumor is more than 2 cm from the Z-line are often treated with WR when they are close to the greater curvature, which can ensure the integrity of the Z-line and will not affect the patency of the cardia. When approaching the lesser curvature, RASW is often used because WR will narrow the cardia and may also lead to excessive gastric wall resection, shorten the length of the lesser curvature, and cause structural malformations or abnormal movements in the stomach. For GISTs in the EGJ in which the upper edge of the tumor is less than 2 cm from the Z-line, mucosa-preserving resection is used to preserve the normal function of the cardia. Zheng et al. (16) reported a new method of conformal resection to treat GISTs in the EGJ in which the upper edge of the tumor is less than 2 cm from the Z-line or has invaded the Z-line, but it needs to be completed by laparotomy. The 2017 edition of “Consensus on Diagnosis and Treatment of Gastrointestinal Stromal Tumors in China” recommends that when GISTs in the EGJ are surgically treated, as long as the upper edge of the tumor is 1–2 cm away from the Z-line and more than 50% of the circumference of the EGJ is retained after the tumor is removed, WR can be used. It can be used to cut the transverse suture under direct vision to avoid narrowing the suture. Proximal gastrectomy was performed for GISTs in the EGJ that invaded the Z-line.

At present, LLG for GISTs in the EGJ in which the upper edge of the tumor is less than 2 cm from the Z-line or has invaded the Z-line <1/2 circumference has not been reported, which presents great challenges in terms of technology and functional preservation. This study analyzed the safety, efficacy and the selection strategy of LLG for GISTs in the EGJ in which the upper edge of the tumor is less than 2 cm from the Z-line or has invaded the Z-line <1/2 circumference.

## Methods

### Patients

This study was a single-center retrospective study. This study was approved by the Ethics Committee of Beijing Friendship Hospital, Capital Medical University. Patients were enrolled: (I) preoperative endoscopic ultrasonography showed mesenchymal tissue-derived tumors, and if the upper margin of the tumor was less than 2 cm from the Z-line or the upper edge had invaded the Z-line and the invasion range was less than 1/2 of the circumference; (II) aged 18–75 years, both male and female; (III) LLG was performed, including WR and RASW, with or without conversion to laparotomy; (IV) no history of gastrointestinal surgery and no history of gastrointestinal malignant tumors; (V) normal organ function; (VI) no found to have distant metastases; and (VII) complete case data and follow-up data. The exclusion criteria were: (I) resection of other organs (liver, pancreas, spleen, or colon); (II) a history of central nervous system disease or mental illness; (III) other diseases that seriously affect survival time; (IV) organ transplantation requiring immunosuppressive therapy; and (V) pregnancy or lactation.

### Surgical techniques

**Figure 1:**
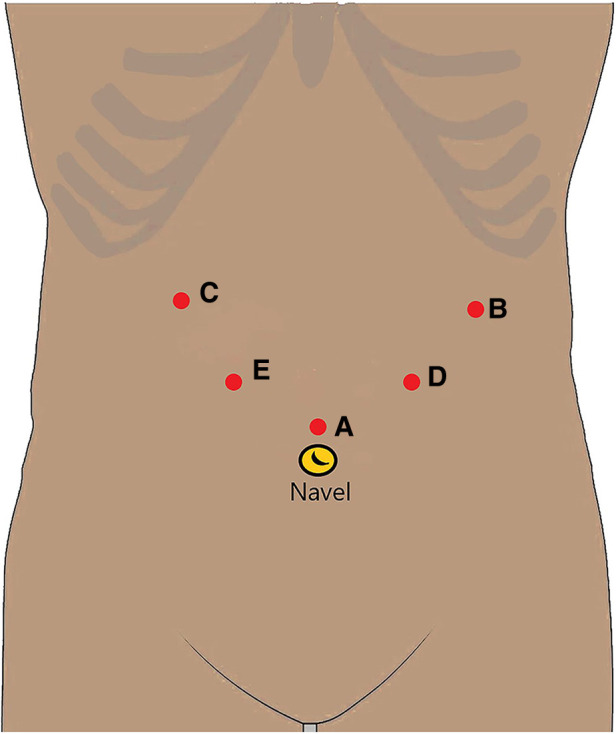
Puncture port placement.

The same group of physicians in the Gastrointestinal Surgery Department performed LLG. The trocar used a 5-hole method (Figure 1). A 12-mm trocar was inserted into the upper edge of the belly button as an observation opening (A). A 12-mm trocar was inserted 2-cm below the costal margin of the left anterior axillary line as the main operation opening for the surgeon (B). A 5-mm trocar was inserted 2-cm below the costal margin of the right anterior axillary line as an auxiliary operation opening for the assistant (C). A 5-mm trocar was inserted 1-cm above the flat umbilicus of the left mid-clavicular line as an auxiliary operation opening for the surgeon (D). A 12-mm trocar was inserted 1 cm below the midpoint of the line between the A hole and the C hole as an auxiliary operation hole for the assistant (E). According to the location of the lesion and the size of the tumor, the surgical method was determined.

**Figure 2:**
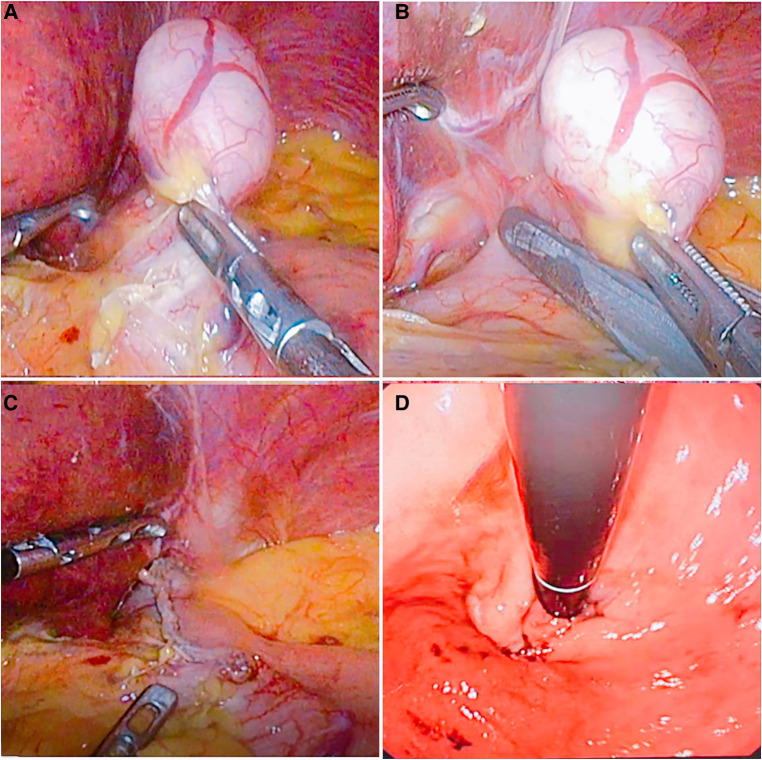
The flow chart of wedge resection. (**A**) The fat and blood vessels on the surface of the stomach in the cardia were opened, exposing the tumor site. (**B**) The tumor was pulled, and a linear stapler was made along the edge of the tumor to remove the tumor. (**C**) The tumor was completely resected. (**D**) Intraoperative gastroscopy was performed to check the patency of the cardia.

**Figure 3:**
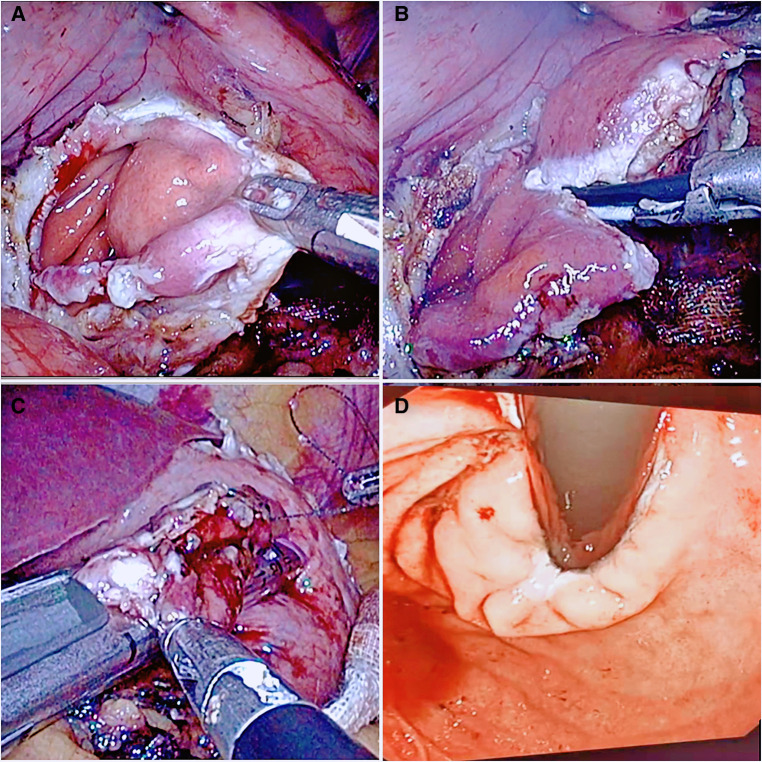
The flow chart of resection by opening all of the layers of the stomach wall. (**A**) The full thickness of the stomach wall was opened to expose the tumor site. (**B**) The tumor was pulled, and an ultrasonic knife was used to remove the tumor. (**C**) After the tumor was completely resected, the cardia was reconstructed using a linear cutting closer. (**D**) Intraoperative gastroscopy was performed to check the patency of the cardia.

(1) WR: for a tumor close to the fundus of the stomach or convex out of the cavity at any position where the upper edge of the tumor is less than 2 cm from the Z-line, insert a 36F thick gastric tube, expand the structure of the cardia, and use a linear stapler to simulate the line to evaluate whether the cardia is stenotic after resection. After assessing that there was no stenosis close to the edge of the tumor, a linear stapler was used to remove the tumor completely (Figure 2).
(2) RASW: if the tumor is located directly below the cardia, close to the lesser curvature, or invades the Z-line circumference and is less than 1/2 or convex into the cavity at any position where the upper edge of the tumor is less than 2 cm from the Z-line, close to the tumor, cut the stomach wall longitudinally at the edge of the tumor throughout the entire process, and entirely remove the tumor along the periphery of the tumor with an ultrasonic knife through the gastric cavity. With the absorbable barb line perpendicular to the long axis of the oesophagus and the esophageal and gastric junction defect, the cardiac structure was reconstructed. Then the plasmic muscle layer was reinforced, embedded, and sutured. Finally, an intraoperative gastroscopy was performed to confirm that there was no stricture of the cardia. A gas-filled water injection test under the gastroscope was performed to verify that there was no air bubble overflow at the anastomosis of the esophagogastric junction (Figure 3).

### Study outcomes

The primary outcome is the incidence of gastroesophageal reflux 1 year after surgery. Full-time staff followed up with patients at the clinic or by telephone, and evaluated the gastroesophageal reflux status with the gastroesophageal reflux disease questionnaire (GerdQ). A GerdQ score ≥8 points indicated gastroesophageal reflux disease. The following data were collected: (I) demographic, including sex, age, and body mass index; (II) auxiliary examination results, including endoscopic ultrasound, and abdominal enhanced CT (III)surgical data, including operation time, blood loss, surgical method, and complications; (IV) postoperative recovery data including time to pass gas, time to oral intake, time to semi-liquid food intake, and postoperative hospital stay; (V) postoperative pathology, including pathological type, tumor diameter and pathological margin; and (VI) the GerdQ of patients before surgery, 6 months after surgery, and 1 year after surgery. Complications were classified according to the Clavien-Dindo classification method (17).

### Statistical analysis

SPSS 21.0 statistical software was used for analysis. The measurement data with a normal distribution are represented by mean ± standard deviation, and values were compared using the independent sample *t*-test. The measurement data with a skewed distribution are represented by median (interquartile range), and values were compared using the non-parametric test. The *X*^2^ test was used to compare countable data. A non-parametric test was used to compare the grade data. Statistical significance was set at *P* < 0.05.

## Results

### Baseline characteristics and pathology

Thirty-eight patients were enrolled from April 2018 to July 2021. Among them, 27 underwent WR, and 11 underwent RASW. The baseline characteristics and pathology of the 38 patients are shown in Table 1. For sex (*P* = 0.579), age (*P* = 0.145), BMI (*P* = 0.512) were comparable between the two groups. There was a significant difference in pathological type (*P* < 0.001) between the two groups. However, in terms of tumor diameter (*P* = 0.406) and pathological margins (*P* = 0.999), no significant between-group differences were observed. All patients achieved adequate R0 margins.

**Table 1:** The baseline characteristics and pathology of the patients.

	WR (*n* = 27)	RASW (*n* = 11)	*P* value
Sex (male/female)	8/19	5/6	0.579
Age (years)	60.5 ± 2.2	54.4 ± 3.6	0.145
BMI (kg/m^2^)	23.8 ± 3.3	24.6 ± 3.3	0.512
Pathological type			<0.001*
Stromal tumor	23	2	
Low/intermediate/high (risk)	14/6/3	1/0/1	
Leiomyoma	4	9	
R0/R1 margin	27/0	11/0	0.999
Tumor diameter (cm)	3.7 ± 1.9	4.2 ± 1.7	0.406

Values are presented as mean ± standard deviation or median (interquartile range). WR, wedge resection; RASW, resection by opening all of the layers of the stomach wall; BMI, body mass index.

* Statistically significant values.

### Perioperative outcomes and follow up

Operative time (90.0 vs. 181.8 min, respectively, *P* = 0.001) was shorter for the WR group vs. RASW. Blood loss (20 vs. 50 ml, respectively, *P* = 0.012) was less for the WR group vs. RASW. Recovery of the RASW group was slower in terms of time to pass gas (2 vs. 3 days, *P* = 0.034), time to oral intake (2 vs. 4 days, *P* = 0.007), time to semi-liquid food intake (4 vs. 8 days, *P* = 0.003), and postoperative hospitalization (5 vs. 8 days, *P* = 0.001) vs. WR. In terms of short-term complications (≤30 days), no significant between-group differences were observed. Cardia stenosis did not occur in either group. No mortality within 30 days of surgery was observed.

Thirty-eight patients were followed up after surgery, and 0 were lost to follow-up. In the WR group, 1 patient experienced mild reflux and scored 11 points at 6 months, which was entirely relieved by taking antacids intermittently. And she was entirely relieved by improving her lifestyle at 1year after surgery, and the GerdQ scored 7 points. In the RASW group, one patient experienced severe gastroesophageal reflux and scored 16 points at 6 months and 1 year after surgery, which was not entirely relieved by taking antacids. No other patients have gastroesophageal reflux. During the follow-up period, there was no death, tumor recurrence, or metastasis (Table 2).

**Table 2:** The perioperative outcomes and follow up of the patients.

	WR (*n* = 27)	RASW (*n* = 11)	*P* value
Approach			0.999
Laparoscopy	27	11	
Laparotomy	0	0	
Operative time (min)	90.0 ± 33.1	181.8 ± 67.7	0.001*
Blood loss (ml)	20 (10–20)	50 (20–50)	0.012*
Time to pass gas (days)	2 (1–3)	3 (2–5)	0.034*
Time to oral intake (days)	2 (1–3)	4 (2–6)	0.007*
Time to semi-liquid food intake (days)	4 (3–6)	8 (5–8)	0.003*
Postoperative hospitalization (days)	5 (3–5)	8 (6–9)	0.001*
Complications (≤30 days)			0.999
Cardia stenosis	0	0	
Anastomotic leakage	0	0	
Anastomotic bleeding	0	0	
Atelectasis	0	1	
Mortality	0	0	
CD grade I/II/III/IV	0/0/0/0	0/1/0/0	
GerdQ (≥8 points)			
Before surgery	6 (22.2%)	4 (36.3%)	0.623
6 months after surgery	1 (3.7%)	1 (9.1%)	0.999
1 year after surgery	0	1 (9.1%)	0.289

Values are presented as mean ± standard deviation or median (interquartile range). WR, wedge resection; RASW, resection by opening all of the layers of the stomach wall; CD, Clavien–Dindo; GerdQ, gastroesophageal reflux disease questionnaire.

* Statistically significant values.

## Discussion

In this study, we used WR and RASW to treat GISTs in the EGJ. For GIST with tumor diameter less than 2 cm, we prioritized endoscopic treatment. However, some patients had difficulty in endoscopic resection under the evaluation of a gastroenterologist, and the risk was high. These patients underwent surgery. The results showed that in the context of ensuring complete resection and negative tumor margins, good results were achieved in terms of cardiac stenosis and gastroesophageal reflux, indicating the physiological anti-reflux function in the EGJ was not completely disrupted. At present, the understanding of the physiological anti-reflux structure in the EGJ is mainly divided into four parts, the lower oesophageal circular muscle sphincter (LECS), upper gastric sphincter (UGS), crural diaphragm with its phrenoesophageal ligament, and gastroesophageal flap valve (GEFV), collectively referred to as the gastro-oesophageal junction high-pressure zone (GEJHPZ) (18, 19). The GEJHPZ functions as a multi-purpose valve. It can regulate the emptying of the esophagus to prevent retrograde reflux of stomach contents while allowing reverse exhaust and retrograde reflux (vomiting). The LECS is a smooth muscle ring in EGJ. It is formed by the noose-like fibers of the upper gastric sphincter, crossing and surrounding the esophagus. The lower boundary is approximately at the level of the His angle, and the upper boundary is approximately 3 cm above the Z-line, where the muscular layer is significantly thicker than in other parts of the surrounding esophagus (18, 20–22). The UGS is composed of gastric sling fibers and clasp fibers. Its acts to reduce the His angle through the contraction of the two fibers, thereby closing the lower end of the esophagus, which has an anti-reflux effect. The lasso fiber starts from the lower part of the lesser curvature and continues diagonally to the upper left corner of His. The lasso fiber is approximately 3-cm wide. Retaining the continuity of the lasso fiber can reduce the occurrence of gastroesophageal reflux. Hook-shaped fibers run horizontally from the muscle bundles located on the side of the minor curve, open to the front left, and are approximately 2.5-cm wide (18, 23, 24). The GEFV is a protrusion formed in the lumen at the His angle of the gastroesophageal junction. It acts like a one-way valve and helps prevent the backflow of stomach contents (18). Therefore, based on the abovementioned anatomical theory, we used the results of preoperative examination and laparoscopy to evaluate the tumor's location to determine the choice of surgery. For tumor close to the fundus of the stomach or convex out of the cavity at any position where the upper edge of the tumor is less than 2 cm from the Z-line, WR is often used. This causes minor damage to the physiological anti-reflux structure and can retain most of the Z-line and surrounding structures. Therefore, WR resulted in shorter operative time, less bleeding, and faster postoperative recovery. However, we need to pay attention to the patency of the cardia during the operation. If the tumor is located directly below the cardia, close to the lesser curvature, or invades the Z-line circumference and is less than 1/2 or convex into the cavity at any position where the upper edge of the tumor is less than 2 cm from the Z-line, WR may lead to stricture of the cardia and destroy more physiological anti-reflux structures. So, we performed RASW. More than 1/3 of the circumference in the EGJ can be retained after the tumor is removed. A longitudinal incision and transverse suture are used to reshape the structure of the cardia and restore the function of the physiological anti-reflux structure as much as possible. At present, based on the research results, our idea is feasible. Therefore, we believe that for GISTs in the EGJ in which the upper edge of the tumor is less than 2 cm from the Z-line or has invaded the Z-line <1/2 circumference, LLG can preserve the physiological anti-reflux function, effectively reducing the incidence of postoperative gastroesophageal reflux and improving the quality of life of patients after surgery.

The anatomy of the EGJ is complex, it is difficult to expose and free the anatomical structure during laparoscopic surgery, and there is a risk of tumor rupture. Therefore, surgeons with extensive laparoscopic experience and skills are recommended to operate, and adequate preoperative evaluations should be performed to reduce the risks and enhance the safety of the operation. Sakamoto et al. (25) found that WR combined with endoscopy for GISTs in the EGJ can improve the safety of the operation. Intraoperative endoscopy plays an increasingly important role in the laparoscopic resection of GISTs, especially GISTs in the EGJ. Endoscopy can not only help to confirm that sufficient surgical margins have been achieved and that the tumor resection is complete but can also check the sutures for stenosis and fistula formation during the operation. Therefore, many scholars consider laparoscopic combined endoscopic surgery essential (26, 27). In our study, WR often used a 36F thick gastric tube to support the cardiac structure, which effectively simulated and evaluated the cardia caliber after resection with a linear closer approach to avoid stenosis of the cardiac opening. For RASW, we used intraoperative endoscopy to evaluate the anastomosis. These two methods provide a safe and effective strategy, contributing to the safety of the operation.

## Conclusion

Overall, it is safe, effective, and feasible to perform LLG for mesenchymal tumors in the EGJ in which the upper edge of the tumor is less than 2 cm from the Z-line or has invaded the Z-line <1/2 circumference. The choice of surgery is based on the relationship between the tumor and the position of the cardia. In the future, it may become the preferred surgical method for mesenchymal tumors in the EGJ. However, the number of cases in this study is small at present. The postoperative follow-up time is still short. In the future, prospective control studies with large samples are needed to evaluate the effectiveness of this procedure further.

## Data Availability

The raw data supporting the conclusions of this article will be made available by the authors, without undue reservation.
